# A correlation comparison between Altmetric Attention Scores and citations for six PLOS journals

**DOI:** 10.1371/journal.pone.0194962

**Published:** 2018-04-05

**Authors:** Wenya Huang, Peiling Wang, Qiang Wu

**Affiliations:** 1 School of Management, University of Science and Technology of China, Hefei, China; 2 School of Information Sciences, University of Tennessee at Knoxville, Knoxville, TN, United States of America; Vrije Universiteit Amsterdam, NETHERLANDS

## Abstract

This study considered all articles published in six Public Library of Science (PLOS) journals in 2012 and Web of Science citations for these articles as of May 2015. A total of 2,406 articles were analyzed to examine the relationships between Altmetric Attention Scores (AAS) and Web of Science citations. The AAS for an article, provided by Altmetric aggregates activities surrounding research outputs in social media (news outlet mentions, tweets, blogs, Wikipedia, etc.). Spearman correlation testing was done on all articles and articles with AAS. Further analysis compared the stratified datasets based on percentile ranks of AAS: top 50%, top 25%, top 10%, and top 1%. Comparisons across the six journals provided additional insights. The results show significant positive correlations between AAS and citations with varied strength for all articles and articles with AAS (or social media mentions), as well as for normalized AAS in the top 50%, top 25%, top 10%, and top 1% datasets. Four of the six PLOS journals, Genetics, Pathogens, Computational Biology, and Neglected Tropical Diseases, show significant positive correlations across all datasets. However, for the two journals with high impact factors, PLOS Biology and Medicine, the results are unexpected: the Medicine articles showed no significant correlations but the Biology articles tested positive for correlations with the whole dataset and the set with AAS. Both journals published substantially fewer articles than the other four journals. Further research to validate the AAS algorithm, adjust the weighting scheme, and include appropriate social media sources is needed to understand the potential uses and meaning of AAS in different contexts and its relationship to other metrics.

## Introduction

Although its original function is retrieval, the applications of a citation index have expanded beyond information retrieval to serve as a tool for bibliometrics and evaluation of scientific merit. In addition to recognizing the quality of reported research, citation also plays a vital role in peer review [1]. An article’s citations reflect its influence. A journal’s citations establish its prestige. Citation, as a quantitative measure, has been used for policy-making, performance evaluation, career advancement, award selection, and funding decisions. However, citation-based metrics have some limitations such as the delay from publication to bibliographic indexing into citation databases [2]. Not all citations are made equal because there are varying reasons to cite an article. A well-known phenomenon is the Matthew effect described by Merton as “the accruing of greater increments of recognition for particular scientific contributions to scientists of considerable repute and the withholding of such recognition from scientists who have not yet made their mark” [3]. Therefore, highly-cited papers are more likely to get even more citations, and so the Matthew effect surely affects the citation behavior [4–6]. In a study to identify factors contributing to the Matthew effect on citations, Wang [7] found weak evidence of any prestige effect. Although they face challenges, citation-based metrics still play a key role in the evaluation of scientific output. Priem [8] believes that citation is an effective way to trace the academic impact of an article. The use of citations continues although alternative measurements are being proposed.

### Alternative approach to traditional citation-based measurements

With the advance of the Web 2.0, especially popular social interaction applications such as LinkedIn, Twitter, and Facebook, Web-enabled reference tools are being developed to help researchers share and manage information. The ways today’s scientists and scholars exchange information and ideas are more diverse and open than ever. Observable information behaviors include not only citations but also accesses, downloads, bookmarks, saves, reads, and recommendations (e.g., tweets). Scientific contributions have been expanded from publishing journal papers and books to actions such as depositing datasets and source code in open access repositories. The traditional citation-based indicators do not measure these social network academic behaviors and the influence of scholars who effectively use Internet-based social networks. AAS is emerging as a new indicator that complements the citation measure of scholarly influence. Priem and associates first coined the term “altmetrics” in 2010 and subsequently published a manifesto [9, 10]. Altmetrics has since garnered great interest. In 2011, the first altmetrics conference was held; in the same year, the International Society for Scientometrics and Informetrics (ISSI) published a special report on altmetrics [11]. Two years later, *Nature* published a comment on the use of altmetrics to track the broad research impact of scientists including all research products, rather than shoehorning everything into article publication [12]. Holmberg and Thelwall [13] investigated disciplinary differences in how researchers used Twitter, the microblogging site. Thelwall et al. [14] discussed the potential value of altmetrics for funding scheme evaluations and conducted a pilot project for the Wellcome Trust. As a result, they provided some important recommendations for future funding stream evaluations, such as selecting a limited number of metrics and comparing articles from the same field.

**Fig 1:**
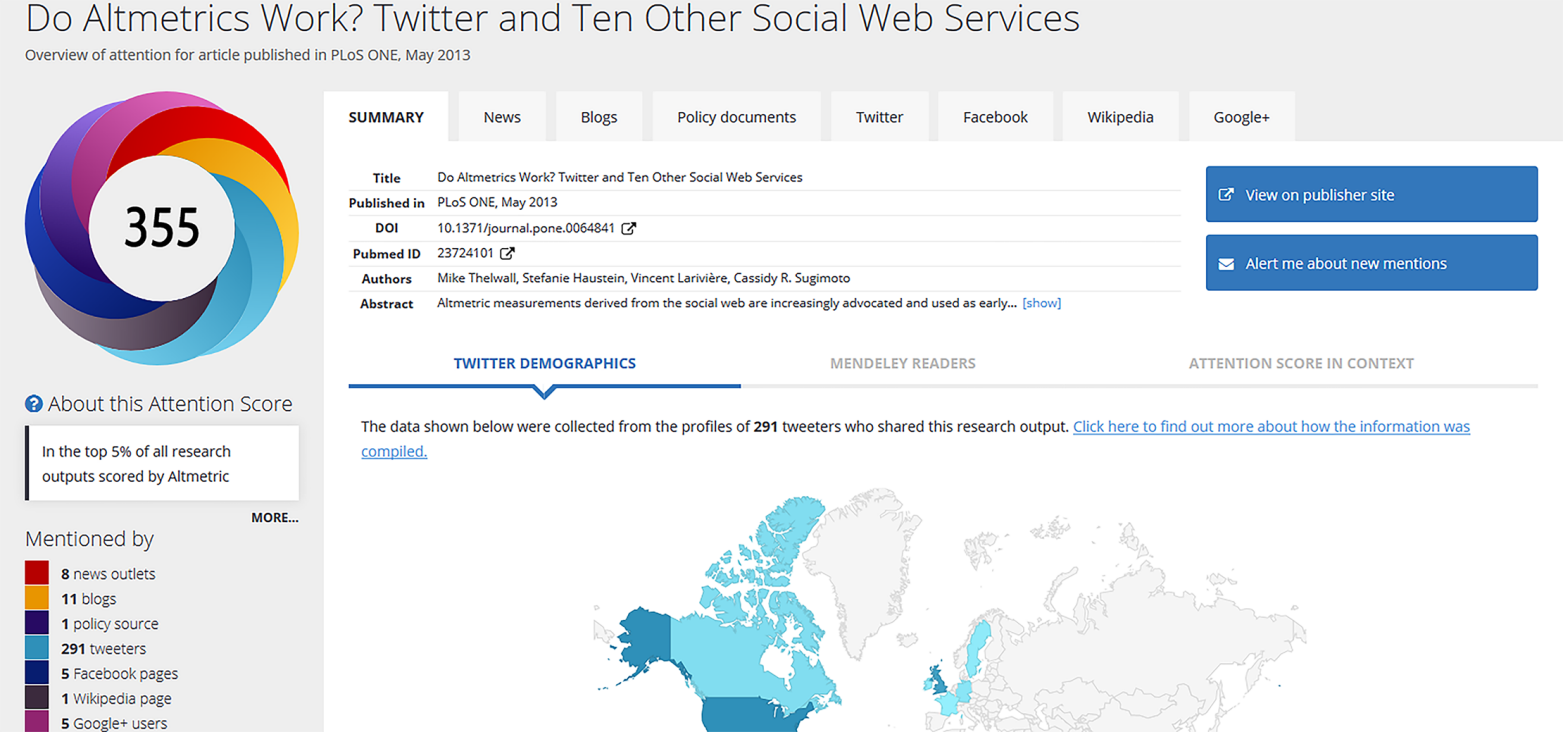
An article’s Altmetric Attention Scores (https://www.altmetric.com/details/6626097) (accessed on May 8, 2016).

The term altmetrics is a combination of *alt*ernative and *metrics*. It measures the interactions of academics, scholars, and scientists as captured by reference management tools and social media such as Facebook, Twitter, LinkedIn, blogs, etc. Many altmetric tools have been developed to track the online activities surrounding scholarly outputs posted to the Web, examples being Altmetric Explore, ImpactStory, ReaderMeter, and Plum Analytics. Altmetric Explore is a tool developed by Altmetric to track article-level metrics for publishers, institutions, researchers, and funders. For article-level metrics, typically the multi-colored altmetric donut badge is found alongside the article: the total score (i.e., Altmetric Attention Score (AAS), also known as Altmetric Score) is in the center, and the breakdown of the individual scores is given as well as the original sources that contribute to the scoring (Fig 1). The authors can also embed altmetric badges of their choice on their personal pages using a free applications programming interface (API). Altmetric maintains a database to track articles through RSS feeds following specific news outlets and blogs. When a publication id is mentioned (e.g., arXiv ids, PubMed ids), the respective mentions are linked to the articles [15]. There are three main goals: (1) to collect as much data as possible about scholarly articles from publishers; (2) to optimize the data for speed and scalability; (3) to provide data sources that can be audited by users [16]. Altmetric, an important altmetric data collector and management company [17–18], provides free access to both the raw data for individual metrics from sources (Twitter, Sina Weibo, Facebook, Reddit, LinkedIn, Google+ users, Mendeley readers, etc.) and the aggregated Scores.

The AAS is derived from the weighted scores of individual indicators (sources) shown in Table 1 by algorithms (not published). The AAS is calculated by summing each indicator multiplied by its weight with necessary adjustments, and the AAS are updated in real-time. The weights are assigned based on the likelihood of the amount of attention which a source may bring to the article. Altmetric has conceptualized altmetrics as “a record of attention” paid to an article in the news, blogs, and on Twitter; as “a measure of dissemination”; and “an indicator of influence and impact.” Altmetric is fully aware of the controversial nature of measuring scientific impact, noting that “It is important to bear in mind that metrics (including citation-based metrics) are merely indicators–they can point to interesting spikes in different types of attention, etc. but are not themselves evidence of such.” (https://www.altmetric.com/about-altmetrics/what-are-altmetrics/)

**Table 1:** Sources and their weights in calculating Altmetric Attention Score^1^.

Sources	Weight
**News**	8
**Blogs**	5
**Wikipedia pages**	3
**Policy Documents (per source)**	3
**Twitter**	1
**Sina Weibo**	1
**F1000/Publons/Pubpeer**	1
**LinkedIn^2^^,^^3^**	0.5
**Q & A^2^**	0.25
**Facebook (public pages)^2^**	0.25
**Video / YouTube^2^**	0.25
**Reddit/Pinterest^2^^,^^3^**	0.25

Note

1. Data are from https://help.altmetric.com/support/solutions/articles/6000060969-how-is-the-altmetric-score-calculated- (modified on 21 June 2016)

2. These scores are rounded up to a whole number at the aggregated level. For example, five, six, seven or eight Facebook posts will have the same score 2.

3. LinkedIn and Pinterest have since been deprecated as sources due to login requirements.

It is not clear when calculating AAS why Altmetric decided to include certain social media but exclude others such as Mendeley readers, an exhaustive and prevalent social media source for scientists [19–21]. The weights assigned to the selected social media have not been theoretically or empirically validated; they seem arbitrary or simply based on the beliefs of the staff of Altmetric. The online documents do not provide the algorithms for aggregation, other than a brief description.

However, ever since its inception in 2010, altmetrics continues to be adopted by authors, publishers, and researchers. At present, both the traditional publishers such as Springer, Elsevier, Taylor & Francis Group, and Nature, and open access (OA) publishers such as Public Library of Science (PLOS, also spelled as PLoS), PeerJ, and F1000 have embedded AAS alongside their articles.

Researchers have conducted studies to understand the relationship between the traditional citation-based metrics and the novel altmetrics [22–33]. Shuai et al. [22], Eysenbach [23], and Peoples et al. [24] found a positive relationship between Twitter activity (Twitter mentions or the number of tweets) and citations. Priem et al. [25] argued for moderate correlations between Mendeley and Web of Science citations, but many altmetric indicators seem to measure impact mostly orthogonal to citation. Li et al. [26] reported that the number of citations from Google Scholar was highly correlated with the number of Mendeley readers. Shema et al.’s results [27] show that the bloggers tend to prefer articles which are more cited than other articles. Thelwall et al. [28] found statistically significant associations between higher metric scores and higher citations for articles with positive altmetric scores in six of the 11 metrics (Twitter, Facebook wall posts, research highlights, blogs, mainstream media, and forums). These researchers did not consider the aggregated AAS.

However, Haustein et al. [29] found a low correlation between tweets and citations, suggesting that these two indicators may not measure the same impact. De Winter [30] also observed that altmetric indicators such as tweets were weakly associated with the number of citations in PLOS ONE, a multi-disciplinary journal. Torres-Salinas et al. [31] reported a weak Spearman coefficient between the number of tweets and citations; and a weak correlation between citations and the number of Mendeley readers or the number of CiteULike readers. Considering the different features of social media, Haustein et al. [32] suggest that altmetrics should be seen as complements to, rather than alternatives to, citations. To investigate the validity of altmetric data, Bornmann [33] observed the categories of the articles recommended in F1000Prime, a post-publication peer recommendation system. He concludes that a normalization of altmetric data is needed in order to measure social impact accurately.

Several studies observed the relationship between the aggregated AAS and citations collecting data from PLOS. You and Tang [34] collected 907 articles from PLOS Genetics, PLOS Computational Biology, and PLOS Biology. They found a significant correlation between AAS and citation counts. Costas et al. [35] studied the relationship between altmetrics (for both individual altmetrics and the aggregated altmetric scores by the authors) and citations. They reported weak positive relationships between the two altmetrics and citations for the entire data set, as well as the highly cited articles. Focusing on journals instead of individual articles, Wang et al. [36] collected data from the seven PLOS journals (PLOS ONE, PLOS Genetics, PLOS Pathogens, PLOS Computational Biology, PLOS Neglected Tropical Diseases, PLOS Biology, and PLOS Medicine). They reported that AAS correlated significantly with the citations for the entire dataset, but the results varied across different years.

In summary, the current body of research focused mostly on relationships between citations and individual altmetric indicators (Twitter, Facebook, Wikipedia pages, Mendeley readers, CiteULike readers, Google+ users, blog mentions, mainstream media mentions, etc.). Only a few studies (e.g. [34], [35], and [36]) have examined the relationship between citations and aggregated altmetrics scores. There is a lack of research on relationships between citations and normalized AAS or variations in contexts, such as disciplinary differences.

### Research -questions

This study is designed to address the following research questions:

Q1. Do the AAS correlate with citations in Web of Science?
Q2. Is there a correlation between high AAS and high citation counts?
Q3. Are there disciplinary differences in correlations between AAS and citation counts?
Q4. Do the AAS indicate the citation numbers for articles?

## Methods

The six PLOS journals (i.e., PLOS Biology, PLOS Medicine, PLOS Computational Biology, PLOS Genetics, PLOS Pathogens, and PLOS Neglected Tropical Diseases) were chosen as the focus of this study for various reasons: (1) They are open access with well-established peer review criteria; (2) Their impact factors are increasing over time; (3) According to the purpose of this study, they have clearly defined disciplines; (4) They are by the same publisher; (5) They are early adopters of the altmetrics method to provide article-level metrics.

To pursue rigor, we adopted several strategies: (1) to exclude PLOS ONE because of subject heterogeneity; (2) to collect all articles published in a specific year for inclusion and comparison; (3) to normalize data to minimize potential effect of journal size; (4) to stratify data by percentile ranks to factor in popularity; (5) to compare datasets across many journals by the same publisher for interdisciplinary comparison.

### Data collection

Data collection was carried out in May 2015. Articles were collected from the six PLOS journals. These journals, representing six disciplinary areas, are appropriate for cross-disciplinary comparisons. PLOS ONE was excluded because of its multidisciplinary nature. The publication year of collected articles was 2012 to allow citations to occur in Web of Science.

The final datasets include 2,406 research articles. For each article, the following data were extracted from the PLOS website: title, author, date of publication, DOI number, and AAS. Citations were from Thomson’s Web of Science and the AAS were provided by Altmetric (https://www.altmetric.com/products/free-tools/bookmarklet/). The Altmetric bookmarklet was installed in the Firefox browser bookmarks toolbar. The following process was utilized for each paper: (1) open a PLOS paper, (2) click on the “Altmetric It” from the toolbar, and (3) get the scores from the popup altmetric donut. (The total altmetric score shows in the middle of the donut graphic.)

### Data analyses

Raw data were imported into SPSS 20.0. Each article has four indicators (Table 2): citations (CIT), Altmetric Attention Scores (AAS), normalized citations (NCIT), and normalized Altmetric Attention Scores (NAAS). To account for the considerable variation across journals (Table 3), the raw data were further normalized for comparison. The field-normalization approach of citations has been used in previous studies [37, 38]. The formulae for normalizing article’s scores in this study are as follows:

NAAS=article'sAAS/meanAASofthejournalinwhichthearticleispublished (1)

NCIT=article'sCIT/meanCITofthejournalinwhichthearticleispublished (2)

**Table 2:** List of indicators.

Abbreviation	Definition
**AAS**	Altmetric Attention Scores from Altmetric-
**CIT**	Web of Science Citations
**NAAS**	Normalized Altmetric Attention Scores by formula (1)
**NCIT**	Normalized Web of Science Citations by formula (2)
**Top AAS 25%** **Top AAS 50%**	The articles stratified in the top 25% set by AAS scores The articles stratified in the top 50% set by AAS scores
**Top NAAS 1%** **Top NAAS 10%** **Top NAAS 25%** **Top NAAS 50%**	The articles stratified in top 1% set by NAAS scores The articles stratified in top 10% set by NAAS scores The articles stratified in top 25% set by NAAS scores The articles stratified in top 50% set by NAAS scores

**Table 3:** Summary of research articles published in six PLOS journals in 2012.

PLOS Journal	Impact Factor	Total	Indicator	Median	Mean	SD	Min	Max
Biology	12.690	134	AAS	7	17.78	37.93	0	358
			CIT	18.5	21.56	15.69	2	89
Medicine	15.253	102	AAS	18	33.80	61.30	1	412
			CIT	14.5	19.64	16.99	0	81
Computational Biology	4.867	466	AAS	2	4.54	9.79	0	112
			CIT	8	10.40	10.93	0	99
Genetics	8.517	682	AAS	2	5.28	13.79	0	243
			CIT	12	16.18	15.49	0	122
Pathogens	8.136	558	AAS	2	4.92	11.18	0	133
			CIT	14	17.50	13.04	0	96
Neglected Tropical Diseases	4.569	464	AAS	1	4.61	39.39	0	845
			CIT	8	10.04	9.12	0	97
*Total*		*2*,*406*	*AAS*	*2*	*6*.*83*	*26*.*04*	*0*	*845*
			*CIT*	*11*	*14*.*63*	*13*.*64*	*0*	*122*

Further, articles are stratified by percentile ranking of NAAS or AAS scores: labeled as top 1%, top 10%, top 25%, or top 50% for comparison. Because this ranking puts tied articles into the same rank, the inclusion of the articles in the top n% is based on the nearest n%. For example, when a journal has more articles tied above the n% (e.g., 50%), the number of articles in the top n_a_% is greater than the number for n% (e.g., 50%) of the total articles for a journal. If a journal has more tied articles below the n%, the number of articles in top n_a_% is less than the number of n%. Thus, the n% is to find the nearest number ~n%.

To quantify the relationships between AAS and citations, Spearman correlations were tested on all articles and articles with AAS. Further analysis compared the stratified datasets based on percentile ranks of AAS: top 50%, top 25%, top 10%, and top 1%. Comparisons across the six journals provided additional insights.

## Results

A total of 2,406 articles were published in the six PLOS specialized journals in 2012 (Table 3). The number of the articles ranges from 102 (Medicine) to 682 (Genetics). The non-normal distributions of AAS or CIT scores for all the six journals show Means > Medians. The variances of AAS among the six journals are noticeably different by a factor of more than six: Medicine has the highest standard deviation (SD = 61.30) while Computational Biology has the smallest SD (9.79). The CIT scores for the six journals are much less varied with less than a twofold difference: the highest SD is 16.99 for Medicine and smallest SD 9.12 for Neglected Tropical Diseases. Both Biology and Medicine have fewer articles than the other four journals, which will be considered in the discussion below.

To rank journals by medians of AAS: Medicine > Biology > Genetics > Pathogens > Computation Biology > Neglected Tropical Diseases. The above list would change the position of the 5th and the 6th if the journal ranking is based on means.

Ranking by CIT scores, means or medians resulted in the same order although the 5th and the 6th journals will tie by median: Biology > Medicine > Pathogens > Genetics > Computation Biology > Neglected Tropical Diseases. The journal rankings based on our data by AAS and CIT seem to be consistent with the journal impact factor (IF) in 2012.

### The PLOS articles with zero AAS or CIT scores

On average, 18.6% the 2012 PLOS six journal articles had not been mentioned in social media as of May 2015 (Table 4). The proportion of articles with zero AAS score ranges from 0.0% to as many as 28.0%. For the same sets of articles, a much smaller percentage (0 ~ 2.9%) of articles had not had citations indexed in Web of Science during the same period. In other words, at least 97.1% of PLOS articles have been cited and indexed in Web of Science after being published for 2.5 years (Table 4). Altmetric began to track online academic activities July 2011. Thus, scientists who have not adopted altmetrics simply might not know of its availability. The PLOS Neglected Tropical Diseases has the highest percentage of articles with zero AAS (28.0%), indicating fewer social media activities about its articles.

**Table 4:** Six journals’ papers with AAS or CIT zero scores.

PLOS Journal	Total	Zero AAS		Zero CIT	
		Number	Percentage	Number	Percentage
Biology	134	5	3.7	0	0.0
Medicine	102	0	0.0	3	2.9
Computational Biology	466	101	21.7	9	1.9
Genetics	682	79	11.6	7	0.1
Pathogens	558	132	23.7	3	0.5
Neglected Tropical Diseases	464	130	28.0	8	1.7
*Total*	*2*,*406*	*447*	*18*.*6*	*30*	*1*.*2*

A close look at PLOS Medicine and Biology shows an interesting phenomenon, which is perhaps unexpected. Both journals took first or second place by IF, AAS, or CIT, but Medicine had the highest percentage of uncited articles (2.9% zero CIT) among the six journals despite its social media activities being the highest (Table 4). For Biology, not all articles had AAS (3.7% zero AAS), but all articles were being cited. Both journals also show high CIT variances (Table 3).

### Testing correlations of normalized data

**Fig 2:**
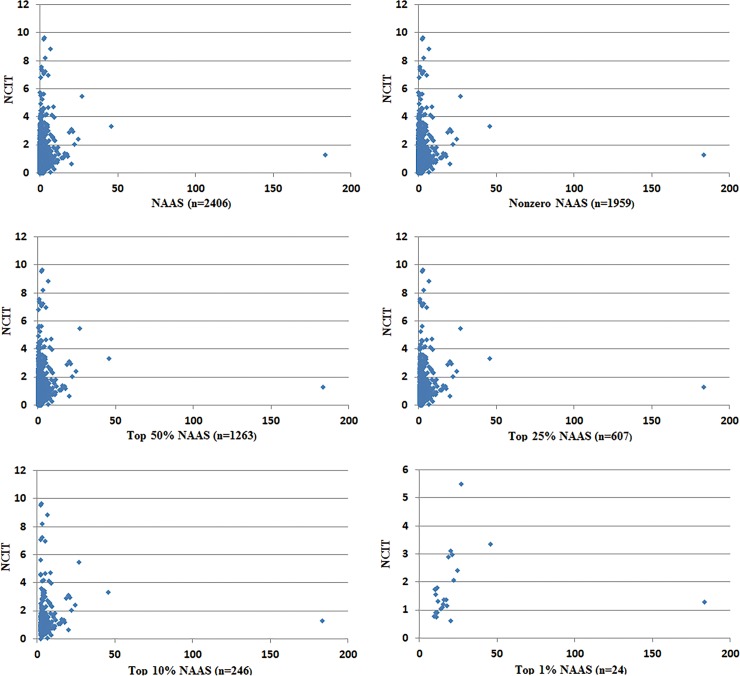
Scatter plots between normalized citations (NCIT) and normalized Altmetric Attention Scores (NAAS).

Normalization was used on the data for correlation analysis to counter the potential effect of differences in size and nature of the journals. Spearman correlation tests of NCIT and NAAS were run on different strata: the whole dataset of 2,406 articles, the 1,959 articles with nonzero NAAS, the 1,263 articles in NAAS top 50%, . . . and the 24 articles in the top 1%. The scatter plots between NCIT and NAAS are shown in Fig 2, and the results of the Spearman correlations are summarized in Table 5. Significant positive correlations were observed between NAAS and NCIT (p < .01) for the entire dataset, the nonzero NAAS dataset, top 50%, top 25%, and top 10%. For the top 1% with only 24 articles, the correlation value is at a relatively weaker significance level (p < .05).

**Table 5:** Spearman correlations between NAAS and NCIT.

Articles	NAAS	Nonzero NAAS	Top 50% NAAS	Top 25% NAAS	Top 10% NAAS	Top 1% NAAS
Number	2,406	1,959	1,263	607	246	24
r_s_	0.227**	0.287**	0.236**	0.200**	0.216**	0.484*

** Significant at 1%

* Significant at 5%

### Testing correlations of individual journals

**Fig 3:**
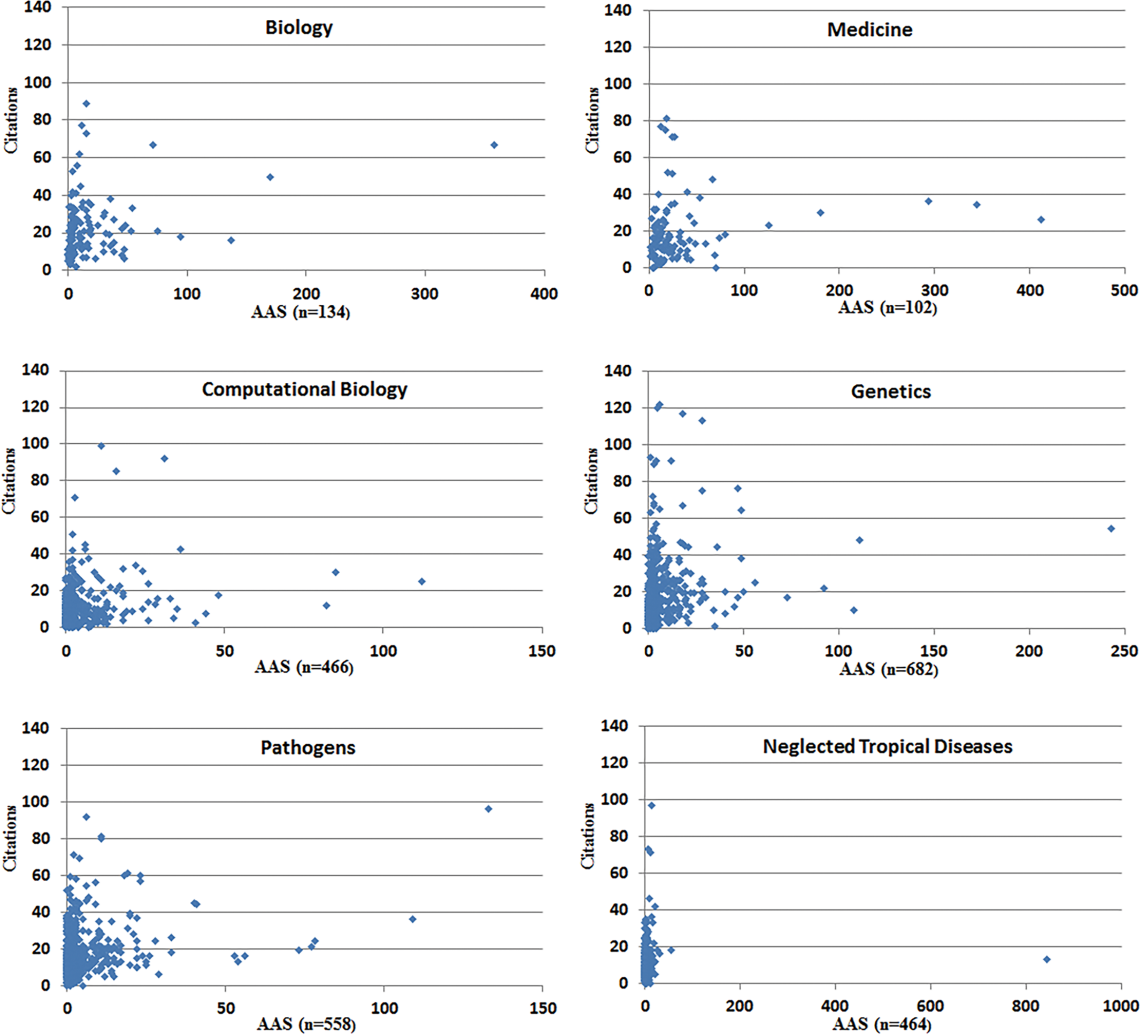
Scatter plots for six PLOS journals.

The Spearman correlations were run for each journal’s datasets: all articles and the nonzero AAS set, as well as the two stratified percentile sets based on AAS: top 50%, and top 25%. Most strata have more tied articles above the rank of n% (see the Methods section for a comment about tied articles). For individual journals, neither the top 10% nor top 1% AAS would have sufficient data, thus they were excluded in the analysis. Scatter graphs of the entire dataset and individual journal datasets show a similar pattern as seen in Fig 3. There is a dense area for lower scored articles. The pattern, although it does not fit a linear model, suggests some relationship between AAS and CIT scores. A few highly-cited articles did not receive high social media attention (top left); a few articles with highest social media attention did not retrieve a high number of citations within 2.5 years (lower right). The results of the Spearman correlations, as presented in Table 6, indicate that four journals show significant positive correlations (p < .01) for three datasets: all articles, articles with AAS, and articles in top 50%. For the dataset of the top 25%, Pathogens shows significance (p < .01) and Genetics, Computational Biology, and Neglected Tropical Diseases show significance at the p < .05 level.

**Table 6:** Spearman correlations for six PLOS journals.

PLOS Journal	AAS		Nonzero AAS		Top 50% AAS		Top 25% AAS	
	Number	r_s_	Number	r_s_	Number	r_s_	Number	r_s_
Biology	134	*0*.341**	129	*0*.290**	71	-0.007	35	0.060
Medicine	102	0.194	102	0.194	52	-0.013	25	0.377
Computational Biology	466	*0*.170**	365	*0*.197**	250	*0*.242**	126	0.212*
Genetics	682	*0*.327**	603	*0*.316**	*369*	*0*.289**	*183*	*0*.169*
Pathogens	558	*0*.216**	426	*0*.216**	293	*0*.228**	*161*	*0*.264**
Neglected Tropical Diseases	464	*0*.156**	334	*0*.234**	212	*0*.276**	134	0.203*

** Significant at 1%

* Significant at 5%

The two top-ranked journals present different pictures. Biology shows a split between the four sets of data: for the entire dataset and the dataset of articles with AAS, significant positive correlations (p < .01) were found, but for the other two sets the Spearman correlations are not significant. None of the datasets for Medicine resulted in significant correlation. Both Biology and Medicine showed small negative values for Spearman correlation, but these values are not statistically significant (Table 6).

Examining the results from another angle by comparing data across columns or rows suggests that as the size of the dataset decreases, the significance values are likely to drop. The smallest journal, Medicine, showed no statistically significant correlation despite being ranked first by AAS and second by CIT. Biology, as the second smallest journal and ranked second by AAS and first by CIT, only showed significant correlations for the entire dataset and the set of articles with AAS. It is also noticeable that by removing the 5 zero AAS articles, the correlation number also decreased although it still shows a significant correlation at p < .05 (Table 6).

## Discussion

This study collected all the articles published in six PLOS specialized journals in 2012 and the Altmetric Attention Scores (AAS) and citations (CIT) from Web of Science as of May 2015 for these articles. The datasets were normalized to validate the calculations including all the journals. Significant positive correlations (p < .01) between the normalized Altmetric Attention Scores (NAAS) and normalized citations (NCIT) were observed for the whole dataset (2,406 articles), the articles with AAS (1,959 articles), and normalized NAAS strata: top 50% (1,263 articles), top 25% (607 articles), and top 10% (246 articles). Papers with higher AAS received more citations except when the journal, such as PLOS Medicine, published relatively few articles. Although the results of this study corroborate the findings by You and Tang [34], it is important to point out the methodological differences between their study and ours: (1) This study normalized raw data to minimize differences across journals of different subject areas; (2) This study focused on the articles published in 2012 to ensure both Altmetric data and citations data had reached a stable point by the time of data collection in 2015; (3) This study analyzed the correlations to several strata based on percentile rank while You and Tang tested their whole dataset of 907 articles from three PLOS journals (Biology, Computational Biology, and Genetics); and (4) This study included six PLOS journals instead of three, and made cross-journal comparisons. Our results advanced understanding of the differences of the three journals studied by [34]: Medicine and Biology have similar ranking by AAS or CIT but different correlations between the two measures. For Medicine articles, no significant correlation was observed. For Biology articles, strong positive significant correlations were observed on the entire dataset of 134 articles and the set of 129 articles with AAS, although a negative non-statistically significant correlation was observed for the set of 71 top 50% articles ranked by AAS.

Four journals, Computational Biology, Genetics, Pathogens, and Neglected Tropical Diseases, were found to have significant positive correlations between AAS and CIT across the datasets (the entire dataset or the stratified datasets). However, for the two highly ranked journals, Biology and Medicine, the results may have been affected by the size of the datasets, in that the only significant correlations were observed for Biology's two datasets, all articles and articles with AAS.

The results from this study corroborate those of Wang et al. [36]. However, these two studies differ in some methodological aspects: (1) This study used Web of Science’s citations while Wang et al. used Scopus’ citations; (2) This study selected the publication year 2012 with a two-year citation window, while [36] collected data from 2004 to 2012; (3) This study excluded PLOS ONE to minimize potential effects of a journal covering multiple disciplines; (4) This study normalized the raw data for journals to minimize the size effect; (5) This study stratified data by percentile ranks to observe differences due to AAS. Therefore, this study observed certain granular differences that have not been reported by other studies. For example, no significant correlation was observed in PLOS Medicine, but both significant and non-significant correlations were observed in Biology depending on the datasets.

Several cautionary notes must be given on the limitations of the conclusions drawn about the altmetric analysis. Altmetrics data are empirical data; thus, there is a lack of theoretical understanding of why and how the research articles are being disseminated through certain social media. Bornmann calls for going beyond correlational analysis to move towards content analysis because his analysis the tweets about Hirsh’s 2005 h-index paper found that 83% of them were perfunctory and only 16% were about methodology and affirmation [39]. Eysenbach warned that unscrupulous researchers could “game” the system by creating more exposure for their work on social media [23]. Critics have also argued about the possibility of manipulating social networks to boost AAS.

It is obvious that the AAS and citations measure different aspects of scholarship. One aspect concerns the dissemination of research outputs through online social media, and one refers to how scientists use these research outputs as documented in their outputs. The scientists who used the research outputs might have found them through different channels. These limitations affect how the altmetric results can be interpreted. Although at its current stage there are certain weaknesses of the AAS as a measure of a scholar's impact, it has obvious popularity among scholars and publishers. Currently, many publishers, such as Springer, Elsevier, Taylor & Francis Group, Nature, PLOS, PeerJ, and F1000, have embedded AAS alongside their publications.

This study used Spearman correlation to identify if there is a significant correlation between AAS and citations. It must be pointed out that although it is a frequently used method in bibliometrics research, correlational analysis in this study provides a simplest first look at how citations and AAS associate in the six PLOS journals. The results did not suggest any causal relationship between them. This is a limitation of our study design. To advance this research, a theoretical framework needs to be developed to identify the many factors underlying citation behavior and social media behavior of researchers. This much needed theoretical framework can guide new data collection for multiple regression analysis to provide more robust results on the effect of AAS on citations.

## Conclusions

To answer the first research question (Do AAS correlate with citations by Web of Science?), the Spearman tests found that normalized AAS correlates with normalized Web of Science citations, but as the scatter plots suggest, there may be different zones of correlations. For the second research question (Is there a correlation between high AAS and high citation counts?), the answer is not simply either Yes or No. There is evidence that higher AAS values are likely associated with high Web of Science citations when the stratified datasets include a sufficient number of articles. The threshold number has not been tested by this study. Further, further stratification in the highly scored articles may show different correlations. The answer to the third research question (Are there disciplinary differences in correlations between AAS and citation counts?) is that differences in correlations between AAS and Web of Science citations across disciplines have been found. PLOS Medicine did not show any significant correlation and PLOS Biology did not show a significant correlation in its rank-based stratified sets. The final question (Do AAS indicate the citation numbers of articles?) must be answered with caution. In general, our empirical results show a possibility that AAS may be an indicator of citation numbers if the nature of the journal is factored in. Thus, it is important to follow up on the fact that journals with fewer articles show weak or no correlations despite their high ranking by AAS, CIT, or IF. Because AAS peaks quickly following publication and then declines over time and because citations in Web of Science are delayed, longitudinal observations are needed to track patterns over time. The journals examined in this study were published in 2012 shortly after the start of altmetrics, when only early adopters were using social media to disseminate research outputs, and this timing issue that perhaps constitutes another factor.

It is also fair to conclude that AAS by Altmetric is a potentially useful measurement of social activities in disseminating research outputs through social media. The attention and awareness brought by social media should hopefully increase the chances for citations. However, scientists cite articles that contribute to their research outputs or for practical reasons such as citing articles by a potential referee [40]. Therefore, it is not surprising that we observed that some articles with higher-than-average AAS do not receive higher-than-average citations. This observation also may suggest that most scientists in 2012 sourced publications through the familiar, traditional channels instead of social media.

Although the results from this study and similar studies indicate that higher AAS are likely associated with higher citations, such correlations cannot suggest a causal relationship. If AAS is used as an impact indicator, it must be differentiated from citation counts or expert evaluation. The AAS should not replace traditional metrics but can provide a wider and deeper view of the social fabric upon which scholars, institutions, funders, and publishers showcase research outputs.

Further studies should explore methods to evaluate different weighting schemes and should include social media which have a potential for impact but are not yet included in Altmetric’s scoring system. Once a rigorous Altmetric Attention Scoring tool is developed and adopted, the challenge will be to integrate it with traditional metrics, such as citations, to construct a comprehensive measurement tool for evaluating research impact.

## Supporting information

S1 Dataset Data used in this study. Data for Six PLOS Journals (PLOS Biology, PLOS Medicine, PLOS Computational Biology, PLOS Genetics, PLOS Pathogens, and PLOS Neglected Tropical Diseases): DOI, Publication month, Altmetric Attention Scores, and Web of Science citations.
(XLSX)
